# Considerations for modelling a broad food tax in the Philippines and other low-income and middle-income countries

**DOI:** 10.1136/bmjgh-2023-012068

**Published:** 2023-10-09

**Authors:** Christine Johnson Curtis, Matti Marklund, Akshar Saxena, Eva Goyena, Josie P Desnacido, Adam D Koon, Bethany Warren, Laura K Cobb, Megan E Henry, Lawrence J Appel, Imelda Angeles-Agdeppa

**Affiliations:** 1 Resolve to Saves Lives, New York, New York, USA; 2 Welch Center for Prevention, Epidemiology, and Clinical Research, Johns Hopkins University, Baltimore, Maryland, USA; 3 Department of Epidemiology, Johns Hopkins University Bloomberg School of Public Health, Baltimore, Maryland, USA; 4 The George Institute for Global Health, University of New South Wales, Sydney, New South Wales, Australia; 5 Department of Public Health and Caring Sciences, Uppsala University, Uppsala, Sweden; 6 School of Social Sciences, Economics, Nanyang Technological University, Singapore; 7 Department of Science and Technology, Food and Nutrition Research Institute, Manila, Philippines; 8 International Health, Johns Hopkins University Bloomberg School of Public Health, Baltimore, Maryland, USA; 9 Division of General Internal Medicine, Johns Hopkins University, Baltimore, Maryland, USA

**Keywords:** Nutrition, Health policy, Prevention strategies

## Abstract

Fiscal policies to improve diet are a promising strategy to address the increasing burden of non-communicable disease, the leading cause of death globally. Sugar-sweetened beverage taxes are the most implemented type of fiscal policy to improve diet. Yet taxes on food, if appropriately structured and applied across the food supply, may support a larger population-level shift towards a healthier diet. Designing these policies and guiding them through the legislative process requires evidence. Equity-oriented cost-effectiveness analyses that estimate the distribution of potential health and economic gains can provide this critical evidence. Taxes on less healthy foods are rarely modelled in low-income and middle-income countries.

We describe considerations for modelling the effect of a food tax, which can provide guidance for food tax policy design. This includes describing issues related to the availability, reliability and level of detail of national data on dietary habits, the nutrient content of foods and food prices; the structure of the nutrient profile model; type of tax; tax rate; pass-through rate and price elasticity. Using the Philippines as an example, we discuss considerations for using existing data to model the potential effect of a tax, while also taking into account the political and food policy context. In this way, we provide a modelling framework that can help guide policy-makers and advocates in designing a food policy to improve the health and well-being of future generations in the Philippines and elsewhere.

**Summary box**

- Fiscal policies for health are a promising strategy to promote public health, while sugar-sweetened beverage taxes are widely adopted, food taxes to promote public health are still rare.
- Food taxes that apply to a wide range of foods, not just sugary drinks, could have greater effects on diet, health and the economy, but the extent of the potential effect is not well known.
- Modelling the potential effects of a broad food tax is limited in low-income and middle-income countries (LMICs).
- Creating a modelling framework that captures the potential effect of food taxes requires reliable input data and knowledge of the political and policy context.
- This study considers how to model the effect of a broad food tax in an LMIC, using the Philippines—where a food tax is currently being considered—as an example.

## Introduction

The increasing availability and affordability of ultraprocessed foods in low-income and-middle income countries (LMICs) has shifted food consumption toward more packaged, processed foods thereby resulting in diets that are high in sugar, sodium and saturated fat.^1^ Poor diet is a modifiable risk factor for non-communicable diseases, which are responsible for almost three-quarters of all deaths globally.^2^ Intake of sugar is related to both overweight and obesity, which are risk factors for non-communicable diseases including diabetes, cardiovascular diseases (CVD), cancer and dental caries.^3^ High sodium intake increases the likelihood of high blood pressure, a key risk factor for CVD, the leading cause of death globally.^4^ High intake of saturated fats, at the expense of unsaturated fats, can also increase heart disease risk.^5^

Fiscal policy, including taxation, is increasingly seen as a way to reduce consumption of less healthy foods.^6^ The WHO recommends taxes on alcohol, tobacco and sugar-sweetened beverages (SSBs) as ‘best buy’ policy measures to change patterns of consumption and improve population health.^7^ The rapid increase in the number of countries implementing SSB taxes shows how politically attractive these taxes have become, particularly in LMICs.^8^ Taxes on a broad range of foods, rather than just SSBs, could potentially have a larger effect on health, but there is limited evidence to date.^6 9^ Taxes on less healthy foods can build on the momentum of SSB taxes to encourage healthier dietary patterns and potentially fund public health efforts that further support healthy diets. Throughout the paper, we will refer to taxes that designate some foods as less healthy, defined by meeting specific nutrient criteria, as ‘food taxes’ for simplicity. A ‘broad’ food tax could target a variety of food categories (eg, breads, snacks, condiments) and consider multiple nutrients of concerns (eg, sugar, sodium).

The process of initiating food taxes is highly politicised and requires persuasive arguments based on evidence. Such evidence, generated by a credible source and using state-of-the-art methods, can provide a compelling rationale for a food tax. Well-designed modelling studies that estimate the potential distribution of health and economic benefits can equip policy-makers and advocates with evidence based on the values of equity, efficiency and welfare. These insights can not only help lead to policy change, but also inform policy design and implementation. Further, the process of modelling can identify gaps in available research and suggest new data collection that will facilitate evaluation of the tax policy once implemented.

The effect of a broad tax on food is rarely modelled in LMICs.^10^ While there are countries with food taxes in place, most are for a small rather than broad set of foods or nutrients (online supplemental table A). Real-world studies of food taxes have demonstrated a decrease in sales of taxed products but not an effect on health, perhaps because of the limited types of food taxed and a longer time frame needed to observe health outcomes.^11 12^ Compared with high-income countries, the data necessary for modelling may be more limited in LMICs, but that should not result in the inequitable application of tax modelling for poorer-resourced settings. As in all modelling efforts, the input data sources and assumptions must be transparent to generate informed and nuanced guidance for policy-makers, advocacy groups and other stakeholders, and to guide future research.

10.1136/bmjgh-2023-012068.supp1 Supplementary data

This paper draws on the authors’ experience as part of an ongoing research collaboration undertaken to support the Government of the Philippines in its efforts to design a food tax, which is currently being considered as a way to encourage healthier diets.^13^ A 2019 report from the Philippines National Tax Research Center considered an excise tax on two categories of foods: snack foods, such as crackers and chips, and fast foods. Based on the revenues of companies that produce these types of foods, the report estimated the tax revenue received at tax rates of 10%, 15% and 20%, but did not use dietary intake data or predict health impact or cost-effectiveness.^14^ The work of our interdisciplinary team, consisting of in-country and international researchers, provides an opportunity to share lessons learnt with the public health nutrition community about how food taxes work and options to consider.

This paper describes issues related to the availability, reliability and level of detail of national data on dietary habits, the nutrient content of foods and food prices; the structure of the nutrient profile model (NPM); type of tax; tax rate; pass-through rate and price elasticity. Using the Philippines as an example, we discuss considerations for using existing data to model the potential effect of a tax, while also taking into account the political and food policy context.

## Key considerations

Table 1 describes key areas and issues for policy-makers to consider when evaluating the potential development and implementation of a food tax, including comments on how these issues apply to the Philippines context. The table is meant to be illustrative and is not comprehensive.

**Table 1:** Considerations for creating a modelling framework

	Key areas	Considerations	Philippines context
Political economy	Political environment	Stakeholder attitudes towards taxation as a tool to promote public health	Stakeholders* include Members of the Governing Board of the Department of Health’s National Nutrition Council, civil society organisations, medical and professional organisations, and the food industry
	Food policy context	a. Readiness for change b. Interaction with existing nutrition policies	a. The Philippines government is actively considering several nutrition policies to promote healthy diets and prevent chronic diseases b. Existing policies include nutrient information on packaged foods; sugar-sweetened beverage (SSB) taxes; trans fat restriction (2023)
	Nutrient profile model (NPM)	a. Consider existing NPM and applicability to taxation	Existing NPMs include the WHO’s Western Pacific Region NPM and South-East Asia Region NPM, both developed for restricting marketing to children
Tax model	Tax model assumptions	a. Type of foods taxed b. Type of tax (eg, excise, value-added tax) c. Tax structure, tax rate and pass-through rate d. Price elasticities e. Sustainability of dietary effect f. Anticipated industry response through reformulation g. Underlying trends in food consumption	a. Packaged foods that are above NPM thresholds b. Excise tax used for SSB tax c. Consider tax rate (~13%) and pass-through data from SSB tax;^39^ conduct sensitivity analysis d. Price elasticities can be calculated from the Philippine’s Food and Nutrition Research Institute price and consumption data e. Conduct sensitivity analysis for sustainability of dietary effects f. Conduct sensitivity analysis for likelihood of product reformulation based on proximity to nutrient threshold g. Food industry sales data
	Model outputs	a. Effect on health (eg, prevented/postponed disease events, prevented/postponed deaths, health-adjusted life years), possibly stratified by socioeconomic status b. Effect on economic measures (eg, healthcare cost savings, government implementation costs, tax revenue, industry reformulation costs) c. Cost-effectiveness (eg, incremental cost-effectiveness ratio, probability of cost-effectiveness).	a. Health-adjusted life-years discount rate (eg, 3%–5%); conduct sensitivity analysis around discount rate b. Costs discounted at the same rate as health-adjusted life-years; net costs calculated depending on perspective c. Incremental cost-effectiveness ratio to be compared with willingness-to-pay thresholds (eg, 1×gross domestic product per capita)^40^
Input data	Dietary and food composition data	a. Foods commonly consumed by the population b. Nutrient profile of food types that are consumed c. Prices of foods	a. The Philippines’ National Nutrition Survey data (2018–2019): single-day dietary recall for 165 225 participants, collected from nationally representative population (subset with 2-day recalls) b. The Food and Nutrition Research Institute packaged food data collected annually for a sample of foods, organised into 18 food categories and over 1600 food codes c. The Food and Nutrition Research Institute data collection for food prices by food code
	Demographics and disease	a. Population size and characteristics (eg, age, sex, socioeconomic status, education, urban/rural) b. Mortality rates, ideally cause-specific c. Risk factor distribution (eg, body mass index, blood pressure) d. Disease epidemiology (eg, incidence, prevalence, case fatality, disability weights) e. Link between diet and disease (eg, dietary effects on risk factors and relative risks for disease)	a. The Philippine Statistics Authority collects demographic and socioeconomic population data through the Labour Force Survey b–d. The Institute for Health Metrics and Evaluation data e. Data from systematic reviews and meta-analyses of randomised trials and large-scale observational studies can provide estimates of dietary effects on risk factors, associations of risk factors with disease risk or direct effects of dietary factors on disease risk.
	Economic data	a. Policy implementation costs (eg, government costs for monitoring and tax collection; industry costs for reformulation) b. Direct healthcare costs (eg, cost per stroke or heart attack, or medication and treatment costs for prevalent cases)	a. Philippines government agency estimates and/or estimates based on the WHO costing tool^41^ b. The Philippines Health Insurance Corporation includes information on healthcare costs; the Philippines Statistical Authority conducts the Family Income and Expenditure Survey and the Philippine National Health Accounts

Three broad areas that may affect a food tax modelling framework are described above. The column on the Philippines context includes relevant organisations, policies and data sources; where data are not available, we have noted that a sensitivity analysis may be used in the modelling framework to test different assumptions.

*Members of the Governing Board of the Department of Health’s National Nutrition Council include the Philippines Government Departments of Science and Technology, Health, Finance, Trade and Industry, Education, Agriculture, Interior and Local Government and the National Economic and Development Authority; civil society organisations include Imagine Law; international partners include WHO, UNICEF and the Global Health Advocacy Incubator; the food industry includes individual companies and associations that represent beverage manufacturers and processed food manufacturers.

## Political economy

### Political environment

The National Nutrition Council (NNC) of the Philippines is responsible for developing, implementing and monitoring food and nutrition policy, including the Philippines Plan of Action on Nutrition. The two departments that are most closely associated with nutrition policy are the Department of Science and Technology-Food and Nutrition Research Institute (DOST-FNRI) and the Department of Health (DOH), which coordinates the NNC (DOH-NNC). DOST-FNRI is responsible for conducting nutritional research, including collection of dietary information through the National Nutrition Survey (NNS) and making policy recommendations. The Department of Finance is responsible for fiscal policy and tax collection.

Civil society organisations have historically been active in lobbying for health policy issues, including increases in the tobacco tax^15^ and passage of the SSB tax to improve public health.^16^ Food and beverage corporations, given their status as regional manufacturers, suppliers and distributors, are highly regarded members of the business community.^17^ The interaction of these actors and, at times, their conflicting priorities, contributes to a highly dynamic food policy environment in the Philippines.^18^ Importantly, the Philippines political environment appears to be receptive to fiscal policy initiatives, thus broad food taxes may be appropriate as a means to further address the rising burden of chronic disease.

### Food policy context

Over the past decade, the Philippines government has put in place protective nutrition policies such as the restriction of unhealthy foods available in schools (updated in 2017)^18^; implementation of a sugary drink tax in 2018^16^; and restriction of industrial trans fat in all foods in the country in 2023.^19^ To accomplish these innovative nutrition policies, the Philippines already had implemented key regulations, such as required back-of-package nutrition labelling for nutrients of concern, including sodium, sugar, saturated fat and trans fat.^20^

Currently, the Philippines legislature is examining a possible food tax and two front-of-package labelling proposals.^13^ DOH-NNC, in partnership with DOST-FNRI, is developing a NPM that will inform future nutrition policies. Concurrently, there are challenges, particularly interference from commercial interests that oppose such nutrition policies. For example, there was a strong resistance from the sugar and beverage industries when the SSB tax was proposed.^21^ In 2019, when DOH raised the possibility of a tax on high salt foods, the Department of Trade and Industry expressed concern about raising the prices of foods that are regularly consumed and the House of Representatives Ways and Means Committee refused to consider it.^22 23^ There is, however, precedent for impactful multisectoral action on tax policy. For example, with government agency and civil society support, in 2012 the Philippines government approved one of the largest cigarette tax increases ever adopted.^15^

## Nutrient profile model

To determine which foods are taxed, policy-makers can reference a NPM, which categorises types of foods according to their nutritional composition, whether it is dietary factors to be encouraged (eg, dietary fibre) or discouraged (eg, sodium).^6^ NPMs can be used to classify foods by producing a score for each food, or NPMs can be used to create a binary distinction of products that are eligible/not eligible for a tax or other policy intervention. NPMs are widely used for nutrition policies such as front-of-package labels, public food procurement standards and marketing restrictions. The WHO Western Pacific Region (WPR) NPM, which was developed for regulating product marketing to children, is provided as an example of a type of NPM (online supplemental table B).^24^ To date, NPMs or single nutrient thresholds are used in only a few food tax policies and have been applied to only a narrow range of food categories.^6^

10.1136/bmjgh-2023-012068.supp2 Supplementary data

To maximise synergy between policies, the same NPM can be considered for complementary nutrition policies. For example, the nutrient thresholds used by Chile to implement a front-of-package warning label system are also used as thresholds that limit marketing to children and restrict the sale of foods in schools,^25^ further incentivising industry to reformulate foods. Using a consistent NPM reduces potential confusion among consumers about healthier food choices.

Table 2 describes considerations for three nutrients of concern that are often included in NPMs, and thus may be targeted by a food tax: sugar, sodium and saturated fat. Although trans fat increases the risk of heart disease, other policy efforts like mandatory trans fat limits or a ban on partially hydrogenated oils have proven effective and may be preferred over taxation. The table gives examples with a single threshold for each nutrient; however, to maximise product reformulation and behaviour change, thresholds may need to be lowered over time or multiple thresholds may be needed per nutrient. In the UK, multiple sugar thresholds for the SSB tax encouraged reformulation.^26^

**Table 2:** Nutrients of concern and related considerations

	Sugar	Sodium	Saturated fat
Health effects of the nutrient	High intake of free sugars* may cause weight gain. In ad libitum studies evaluating effects on body weight of either increased or decreased intake of free sugar, higher sugar intake was associated with about 0.8 kg weight gain.^42^ Weight gain can lead to increased disease risk, one of the top three metabolic risk factors for death and disability globally.^43^ Intake of free sugars is also associated with dental caries.^3^	High sodium intake is the leading dietary risk factor for deaths globally.^44^ Reducing sodium intake lowers blood pressure (on average 2.4 mm Hg lower systolic blood pressure per 1 g reduction of dietary sodium).^45^ High blood pressure is the leading risk factor for preventable cardiovascular disease.^46^	Replacing saturated fat with unsaturated fatty acids (especially polyunsaturated fatty acids) can improve blood cholesterol levels and reduce risk of cardiovascular disease. A meta-analysis of four trials suggested that such replacement lowered coronary heart disease risk by 29% (95% CI 19% to 39%).^47^ The effect on health of replacing saturated fat with carbohydrates appear to depend on the type of carbohydrates (eg, fibre-rich whole grains vs starch-rich refined grains or free sugars).^47^
Nutrient Informa-tion Needed on Packaged Foods	Sugar content is required on nutrition information panel; added sugar content is preferred, but usually only total sugar is available, which can be used as a proxy.	Sodium content is required on the nutrition information panel.	Saturated fat content is required on the nutrition information panel.
Potential Sources of Nutrient Thresh-olds	Regional WHO Nutrient Profile Models (NPMs) include sugar thresholds for relevant categories.	Regional WHO NPMs include sodium thresholds for relevant categories; WHO Sodium Benchmarks set maximums for 60+ categories;^48^ countries may have sodium targets.	Some NPMs have saturated fat thresholds;^49^ or appropriate thresholds may be determined through analysis of labelled saturated fat content by packaged food category.
Tax Policy Examples: Nutrient Limits for Food	Hungary: Prepackaged products with added sugar >25 g sugar/100 g; Chocolates and sugar sweetened cocoa powder: >40 g sugar/100 g and <40 g cocoa/100 g; Fruit preserves/jam: >35 g sugar/100 g^50^	Hungary: Salted snacks >1 g salt/100 g; Condiments (with some exemptions) >5 g salt/100 g^50^	Denmark: Set, then repealed, a tax on products containing >2.3 g saturated fat/100 g of fat; food categories included meat, dairy products, edible oils and fats (with some exemptions).^51^
Analysis considerations	Track use of non-caloric sweeteners to replace sugar, particularly in products targeted at children;^52^ most evidence on sugar reformulation is from sugar-sweetened beverage taxes	Discretionary salt intake is often not captured using current dietary assessment methods.^53^	Consider different ratios of substitution with various types of fats and/or carbohydrates.

Food taxes could be used to discourage consumption of foods that are high in certain nutrients, such as sugar, sodium and saturated fat. These nutrients are listed above, along with nutrient-specific considerations relevant to taxation.

*WHO defines free sugars as “monosaccharides (such as glucose, fructose) and disaccharides (such as sucrose or table sugar) added to foods and drinks by the manufacturer, cook or consumer, and sugars naturally present in honey, syrups, fruit juices and fruit juice concentrates.”

In the absence of an established NPM, the modelling framework could use the WPR NPM as a starting point and, where needed, the South-East Asia Region NPM to consider appropriate thresholds for nutrients of concern.^27^ These NPMs were created to restrict marketing to children, so they will need to be adapted to be appropriate for the Philippines context and for tax policy. As is common, the Philippine food composition table does not contain information on added sugar. Total sugar thresholds can be used, assuming that reformulation to reduce total sugar content would primarily be through reductions in added sugars. Modelling changes in saturated fat intake is complex: the effect on health depends on the type of fat or carbohydrate substituted for saturated fat, the type of saturated fat and the type of saturated fat-rich food considered (eg, palm oil, red meat, cheese or yoghurt).^5^ Most foods in the Philippine food composition table do not contain information on saturated fat content. As a result, it may not be possible to model the effect on saturated fat intake, or any downstream health effects.

## Tax model design

While the goal of a food tax is population-wide improvements to diet and health, the tax should be designed to reflect the country context (table 1). We describe below the key aspects of a modelling framework for estimating dietary, health and economic effects of food taxes in an LMIC; we also describe how to adapt the framework to the Philippines context.

### Tax type

The tax can be structured as a Value-Added Tax (VAT) or an excise tax. VAT is applied as a percentage of the value of the product, whereas an excise tax can be applied as a percentage of the value of the product based on a physical attribute of the product, such as product volume (eg, 100 mL of a beverage) or content (eg, 25 mg of sugar). While WHO does not recommend a specific tax structure, recent research supports using excise taxes,^28^ which is the approach we will use for the Philippines modelling framework.

### Taxable foods and food sources

Proposed criteria for foods that are eligible for a tax could be packaged food with a nutrition information panel that exceeds one or more nutrient thresholds set by the NPM. Certain foods may be exempt from the tax, due to political or cultural reasons. The NPM, including the nutrients chosen, threshold levels or scores, and number of thresholds per nutrient, will affect the scope and impact of the food tax.

To model the potential effects of a food tax, we will use data from the NNS, which collects population dietary intake in the Philippines. Data from the 2018–2019 NNS make it possible to quantify pretax intake by food source, simplifying identification of potentially taxable foods.

### Tax rate and pass-through rate

A food tax modelling framework can assess the effects on diet, health and the economy, using multiple tax rates to inform policy development. For context, WHO recommends increasing the retail price of SSBs by at least 20%.^29^ In the Philippines, the National Tax Research Center produced a 2019 report that examined a potential food tax of 10%–20%. Similarly, while data on the pass-through rate (ie, how much of the tax is paid by the consumers, and not manufacturers or retailers) could be informed by experience from the Philippines SSB tax, the impact of alternative assumptions can be explored through sensitivity analyses, such as comparing a 100% pass-through rate with 50% and 150% pass-through rates. While the pass-through rate greatly influences both health impact and costs, a previous modelling study of an SSB tax in the USA, suggested that it will have limited effect on the cost-effectiveness of the policy (as assessed by the incremental cost-effectiveness ratio).^30^

### Consumer response to price changes

To understand how consumers will respond to changes in food prices, the price elasticity of individual foods or whole food categories is calculated. Own-price elasticity measures the change in consumer demand in response to a change in the price of a product. In our preliminary analyses using NNS data, the own-price elasticities for different groups of packaged foods in the Philippines range from −0.33 for chocolates to −1.8 for cheese spreads and related items, suggesting that a 20% tax rate could reduce intake of taxed chocolates by 6.6% and taxed cheese spreads by 36%.

Using the NNS data, we can also estimate cross-price elasticities, which can help determine the change in consumer demand of other products as the price of a product changes. While NNS data does not report change in consumption over time in response to a price change for an individual or household, and there may be underreporting of intake, we can estimate own-price and cross-price elasticities of demand for groups of similar products.^31 32^ This information can help model the tax impact on food consumption and expenditure and, by extension, the health impact and burden of taxes (as a proportion of income) borne by individuals.

### Sustainability of dietary effect

In a tax modelling framework, we assume in the primary analysis that a tax-induced food price change would instantaneously impact consumption and that this effect is maintained over the period the tax is enforced. Poorer households, who spend a relatively higher proportion of their income on food purchases, may spend more on processed food and may be constrained in their ability to switch to non-taxed substitutes. Hence, the tax is likely to form a larger proportion of household income in the short term; however, over the lifetime of the household members, the induced reduction in consumption of unhealthy food is likely to accord them the greatest health benefits from the tax, taking into account reduced disease burden and lower healthcare costs.^33^ For modelling the tax impact on health and the economy, we can also evaluate alternative assumptions regarding the sustainability of dietary effects in sensitivity analyses.

### Anticipated industry response through reformulation

The way the tax is structured can drive reformulation of products, as companies change product formulations to avoid taxes. If only one threshold per nutrient is defined, we would assume that products close to (but still exceeding) the threshold are most likely to be reformulated to avoid taxation. Products grossly exceeding the threshold (eg, more than 50% or 100% above the threshold) may be less likely to be reformulated, and there would be no incentive to reformulate products below the threshold. Assumptions about reformulation may be informed by the recently implemented SSB tax and can also be tested in sensitivity analyses.

### Underlying trends in food consumption and risk factors

Globally, and especially in LMICs, there is an ongoing trend of increased consumption of processed foods.^1^ In the Philippines, sales data indicate that purchases of highly processed foods per capita have increased since at least 2014.^34^ Even if a food tax may have a small immediate effect on intake, the upward trend in processed food consumption suggests that food taxes could have a larger impact in the future. Such trends, estimated from sales data, can help predict future consumption levels with and without the tax.

## Data

The availability and reliability of input data affects the quality and policy relevance of model outcomes. As part of the modelling framework, we will estimate effects on diet, health, and economy, as well as the cost-effectiveness of the tax.

### Dietary and food composition data

To estimate the dietary impact of the tax, information regarding pretax dietary intake of the target population, overall or stratified by factors like sex, age, socioeconomic status and region, is necessary. Ideally, dietary intake data should be valid, contemporary and representative of the target population. Although true dietary intake is difficult to measure due to reporting biases and inaccuracies in food composition data, repeated 24-hour dietary records or recalls are often considered adequate proxies.

In the Philippines, the NNS assesses food consumption both at the individual and household levels. Data on individual food consumption is estimated using single 24-hour recalls, with a subgroup of participants providing data from 2-day, non-consecutive 24-hour recalls.^35^ The household-level food consumption assessment includes food weighing, in combination with a household food inventory of non-perishable food items such as condiments and table salt.

There are a few common data limitations that can arise in estimating current intakes and predicting future dietary intakes. First, potential misreporting of dietary intake should be investigated and addressed to minimise the risk of inaccurate estimates of habitual intake. While there are multiple ways to address this issue,^36^ NNS data allow for the use of the Goldberg-cutoff method, which defines individual-level thresholds determined by a set of health and physical activity factors.^37 38^ As an example, using single-day 24-hour recalls from the 2018–2019 NNS data, the mean reported energy intake among adult (18+ years) women in the Philippines was 1387 kcal/day, which increased to 1704 kcal/day after the exclusion of potentially misreporting participants identified by the Goldberg-cutoff method.

Second, the categorisation of foods in the dietary intake survey should be aligned with the food categorisation of the NPM underlying the tax, to estimate how the tax will influence dietary intakes. While NNS data do not provide product-level data, it reports information by food codes, which group similar foods together; multiple food codes make up food categories. Modelling dietary intake using food codes allows the calculation of reduced intake of taxed foods (ie, consumer response to changes in price) and reduced content of targeted nutrients (eg, sugar, sodium) in taxable foods through product reformulation.

Third, nutrient and energy content of the food composition tables can be outdated and may not contain full data on some nutrients that could be of interest for a food tax (eg, saturated fat, added sugar). If sodium and total sugar content are available, as they are in the Philippines, the model can focus on the health effects of addressing two nutrients of concern.

Fourth, dietary recall data may not capture all intakes well (eg, discretionary salt). This is a challenge for sodium intake estimation and also price elasticity calculations. If the price elasticities of or between such food categories cannot be calculated, estimates from other populations and settings may be necessary.

### Other considerations

Implementation of food taxes will generate revenue, which can further support a healthy diet by subsidising healthier foods or supporting programmes that improve public health. The model can also estimate changes in fruit and vegetable intake after the tax is implemented, particularly in poorer households, which may help inform policy.

## Conclusion

To our knowledge, this project, led by Johns Hopkins University and the DOST-FNRI in the Philippines with support from Resolve to Save Lives, is the first to develop a model that will assess the cost-effectiveness of a broad food tax in an LMIC. The current political and food policy context in the Philippines appears conducive to a broad food tax that supports healthier diets. While there are some data limitations, which will require the model to rely on assumptions, the available data are detailed enough to develop a Philippines-specific food tax modelling framework which can inform the design of a food tax and subsequent policy discussions.

This approach is likely relevant to other LMICs, where dietary intake and packaged food nutrition data are sometimes limited. A useful model for a broad food tax can provide an estimate for change in nutrient intake, health effects and economic effects, including revenue generation and healthcare cost savings at different tax rates and pass-through levels. This information can guide government agency policy proposals, inform plans for potential revenues and identify issues that need to be addressed, such as subsidies or support for people who will be most affected by the increase in food prices. In this way, carefully planned modelling research can help shape policy to support healthier food environments in LMICs.

## Data Availability

Data available upon reasonable request.
